# Adherence to antiretroviral therapy (ART) during the early months of treatment in rural Zambia: influence of demographic characteristics and social surroundings of patients

**DOI:** 10.1186/1476-0711-11-34

**Published:** 2012-12-28

**Authors:** Yuri Sasaki, Kazuhiro Kakimoto, Christopher Dube, Izukanji Sikazwe, Crispin Moyo, Gardner Syakantu, Kenichi Komada, Shinsuke Miyano, Naoko Ishikawa, Kiyoshi Kita, Ichiro Kai

**Affiliations:** 1 Department of Infection Control and Prevention, Graduate School of Nursing, Nagoya City University, Kawasumi 1, Mizuho-ku, Nagoya-shi, Aichi, 467-8601, Japan; 2 School of Nursing, Osaka Prefecture University, 3-7-30, Habikino-shi, Osaka, 583-8555, Japan; 3 Mumbwa District Health Office, P.O.Box 830018, c/o Mumbwa, Zambia; 4 Ministry of Health, Ndeke House, P.O.Box 30205, Lusaka, Zambia; 5 Japan International Cooperation Agency (JICA), Nibancho Center Building 5-25, Niban-cho, Chiyoda-ku, Tokyo, 102-8012, Japan; 6 Department of International Medical Cooperation, National Center for Global Health and Medicine (NCGM), 1-21-1 Toyama, Shinjuku-ku, Tokyo, 162-8655, Japan; 7 Graduate School of Medicine, The University of Tokyo, 7-3-1 Hongo, Bunkyo-ku, Tokyo, 113-0033, Japan

## Abstract

**Background:**

Around 70% of those living with HIV in need of treatment accessed antiretroviral therapy (ART) in Zambia by 2009. However, sustaining high levels of adherence to ART is a challenge. This study aimed to identify the predictive factors associated with ART adherence during the early months of treatment in rural Zambia.

**Methods:**

This is a field based observational longitudinal study in Mumbwa district, which is located 150 km west of Lusaka, the capital of Zambia. Treatment naive patients aged over 15 years, who initiated treatment during September-November 2010, were enrolled. Patients were interviewed at the initiation and six weeks later. The treatment adherence was measured according to self-reporting by the patients. Multiple logistic regression analysis was performed to identify the predictive factors associated with the adherence.

**Results:**

Of 157 patients, 59.9% were fully adherent to the treatment six weeks after starting ART. According to the multivariable analysis, full adherence was associated with being female [Adjusted Odds Ratio (AOR), 3.3; 95% Confidence interval (CI), 1.2-8.9], having a spouse who were also on ART (AOR, 4.4; 95% CI, 1.5-13.1), and experience of food insufficiency in the previous 30 days (AOR, 5.0; 95% CI, 1.8-13.8). Some of the most common reasons for missed doses were long distance to health facilities (n = 21, 53.8%), food insufficiency (n = 20, 51.3%), and being busy with other activities such as work (n = 15, 38.5%).

**Conclusions:**

The treatment adherence continues to be a significant challenge in rural Zambia. Social supports from spouses and people on ART could facilitate their treatment adherence. This is likely to require attention by ART services in the future, focusing on different social influences on male and female in rural Zambia. In addition, poverty reduction strategies may help to reinforce adherence to ART and could mitigate the influence of HIV infection for poor patients and those who fall into poverty after starting ART.

## Background

Sub-Saharan Africa contains nearly 70% of the world’s HIV infections. In 2009, the average number of people living with HIV (PLWH) reached 22.5 million [1-3]. Zambia is one of the most severely affected countries in the region. Since Zambia’s first reported AIDS diagnosis in 1984, the proportion of PLWH has rapidly increased, peaking in the mid-1990s at about 16% and reaching as high as 25% in some urban areas [4,5]. The Zambia HIV epidemic has moderately stabilized over the last 15 years with a very modest decline after the initial peak in prevalence [6]. Although the rate of new HIV infections has decreased, the total number of PLWH continues to rise. An estimated average of 980,000 (890,000–1,100,000) people in a population of 12.9 million have been infected with HIV in Zambia [6,7]. Adults aged 15–49 had an HIV prevalence of 13.5% in 2009, which was the 6th highest in Sub-Saharan countries [2].

The National HIV/AIDS/STD/TB Council (NAC) in Zambia became operational in 2002. One of its key priorities is the provision of care, treatment and support to PLWH [3]. In 2004, the Ministry of Health (MoH) in Zambia offered antiretroviral therapy (ART) at four clinics in Lusaka, the capital of Zambia. The government declared that the entire ART service package would be provided free of charge in the public sector, with a goal of universal access to HIV care and treatment [8].

As more than half of the population lives in rural areas where there is poor access to health services [9], the MoH aimed to develop approaches to expand services by strengthening the existing public health care system and expressed its intention to expand HIV testing and treatment facilities to all districts and as close to households as possible [10]. With this effort, 283,863 (68%) people out of an estimated total population of 416,533 who were in need of ART received it at 447 health facility sites throughout the country by 2009, and the number of sites is being expanded further [6]. While achievements have been remarkable, the universal coverage and retaining the patients on ART remain as challenges. Studies showed that 59.5% of patients in Zambia’s southern province throughout a period of 15–723 days (a median follow-up of 275 days) and 62.9% of patients in Lusaka over the first 12 months (a median follow-up of 15.7 months from 12 months onwards) were adherent to ART[11,12].

Some reports also have revealed the factors associated with adherence in both urban and rural settings [8,12-18]. The common reasons raised by patients on ART for poor adherence in several qualitative studies included demographic and physical health factors (e.g. insufficient food and side effects) as well as interpersonal factors (e.g. lack of support) [15,16,18]. Other barriers were patients’ mental health factors (e.g. fear of stigma/disclosure and presence of depression/ hopelessness) [16,18].

Such qualitative studies in Zambia generate information from the respondent’s perspective that may facilitate culturally appropriate and effective interventions. However, few quantitative studies have investigated associations between the treatment adherence and factors identified in these qualitative studies. In addition, there is a paucity of studies which have investigated factors related to the treatment adherence during the early months of treatment. Since most complications including deaths occur within early period of treatment [8], optimizing adherence during this stage is important for ensuring long-term immunological and virological treatment success [19,20].

Thus, the objective of this study was to identify predictive factors associated with ART adherence during the early months of treatment in rural Zambia so as to propose possible interventions for future treatment strategies in this region.

## Methods

### Study design and study site

A field based observational longitudinal study was conducted in Mumbwa district, which is located 150 km west of Lusaka. There were one district hospital and 27 rural health centers in Mumbwa during the study period. Among the health facilities, ART services were available at the district hospital, and at eight rural health centers.

### Study population

**Figure 1:**
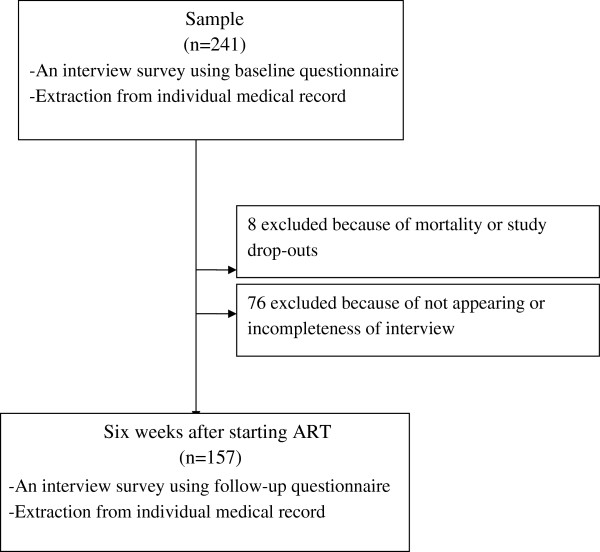
Description of study sample.

During the study period, all patients who met the criteria of treatment initiation according to the Zambia HIV National Guidelines and visited the district hospital or one of the eight rural health centers were invited to participate in the study. The eligible population for this study comprised patients who 1) were aged 16 and over; 2) were ART-naïve and newly registered for ART services from September to November 2010; and 3) agreed to give informed consent. The exclusion criteria included patients who were too ill to be interviewed. Patients were interviewed at the initiation of treatment as a baseline and six weeks later as a follow-up (Figure 1).

### Study tools

Structured questionnaires for both baseline and follow-up interviews were developed, following the generic tools developed by World Health Organization (WHO) [21] and the AIDS clinical trial group (ACTG) adherence follow-up questionnaires [22]. The questionnaires were first developed in English. After being translated into Nyanja (dominant regional language), these were back-translated to English to ensure its clarity and consistency. The questionnaires covered respondents’ sociodemographic characteristics, ART adherence, disclosure status, physical and mental health related characteristics. WHO HIV/AIDS stage [23], weight, CD4 cell count, and Tuberculosis (TB) status were extracted from individual medical records.

In this study, full adherence to ART was defined as when a patient had never skipped prescribed drugs and had followed time restrictions during the previous four days before the interview. Scores for internalized AIDS-related stigma and cognitive/affective depression were measured using scales developed and validated by Kalichman [24]. To simplify the administration, the items were responded to dichotomously, 1=agree and 0=disagree; and scale scores represent the sum total of endorsed stigma items, range 0–6. The cognitive/affective depression subscale is an 11-item measure that assesses symptoms of depression over the previous seven days, 0=no days, 1=1-2 days, 2=3-4 days, and 3= 5–7 days. The median of each scale score was used as the cutoff point between patients who had self-stigma or depressive symptoms and who did not.

Food insufficiency was measured by using one closed-ended question following the Sociodemographic Module of the Client Instrument developed by WHO as a generic tool for operational research on HIV testing, treatment and prevention [21]. Patients were asked to recall the frequency with which there was not enough food in the month prior to interview: never, sometimes, often, or almost always. A similar single question assessment of food insufficiency has been validated in previous studies [25,26].

### Data collection

Before the commencement of the study, a three-day training course on research protocols, administration of questionnaires, and ethics was conducted for eight interviewers. Then field surveys were carried out from September 2010 to March 2011.

### Data analysis

Data obtained from the questionnaire surveys were analyzed with SPSS version 19 statistical software. Baseline characteristics of participants were compared between patients who were adherent to ART six weeks after starting the treatment and those who were not, using Pearson’s chi-square test and Fisher’s exact test. Multiple logistic regression analysis was performed to identify the predictive factors associated with ART adherence. The variables of which the associated *p* value level was less than 0.1 were entered into the multiple logistic regression model. If the variable was highly correlated with the other variable, one of them was removed from the model. An adjusted odds ratio (AOR) was calculated for the levels of the other factors included in the model.

### Ethical considerations

This study was approved by the Research Ethics Committee of the University of Tokyo, the Biomedical Research Ethics Committee of the University of Zambia, and the Institutional Ethics Committee of the National Center for Global Health and Medicine (NCGM). Written informed consent was obtained from respondents at the beginning of the interview, after the study was explained to them. They were informed that participation in the study was voluntary.

## Results

### Description of study sample

During the field research, 241 patients who were newly registered for ART services were enrolled and interviewed enrolled and interviewed (Figure 1). After the baseline survey, 84 could not be interviewed six weeks later because six patients had passed away, two patients referred to the other health facilities, and 76 patients did not answer questions completely at the baseline or did not appear on the appointment date and we could not reach their contact address. The sociodemographic, physical and mental health of these patients did not differ from the patients included in the analysis except for the required time to reach the health facilities (Table 1). Therefore, data from the remaining 157 were used for statistical analysis.

**Table 1:** Characteristics of patients who were excluded compared with those included in the study

	**Excluded**	**Included**	
	**(n=84)**	**(n=157)**	**p-value**
	**n(%)**	**n(%)**	
Site			
Mumbwa District Hospital	42 (50.0)	74 (47.1)	0.671
Rural Health Centers	42 (50.0)	83 (52.9)	
Age			
<35 years old	40 (47.6)	70 (46.1)	0.817
≥35 years old	44 (52.4)	82 (53.9)	
Sex			
Female	43 (51.2)	94 (59.9)	0.195
Male	41 (48.8)	63 (40.1)	
Education			
No or primary incomplete	39 (46.4)	77 (49.4)	0.665
Primary complete or more	45 (53.6)	79 (50.6)	
Marital status			
Married	46 (55.4)	105 (66.9)	0.080
Not married	37 (44.6)	52 (33.1)	
Occupation			
Agriculture	49 (58.3)	107 (68.2)	0.128
Others	35 (41.7)	50 (31.8)	
Transportation			
On foot	50 (59.5)	80 (51.0)	0.204
Others	34 (40.5)	77 (49.0)	
Time required for transportation to health facilities by above method			
Within one hour	51 (61.4)	70 (45.2)	0.017
More than one hour	32 (38.6)	85 (54.8)	
Transportation cost			
Zero	72 (85.7)	127 (80.9)	0.347
More than zero	12 (14.3)	30 (19.1)	
Lack of food during the past 30 days			
Yes	27 (32.5)	61 (38.9)	0.334
No	56 (67.5)	96 (61.1)	
Functional status			
Working	69 (85.2)	138 (87.9)	0.556
Ambulant or bed lid	12 (14.8)	19 (12.1)	
Tuberculosis			
Positive	9 (11.1)	11 (7.1)	0.286
Negative or not sure	72 (88.9)	145 (92.9)	
Self-stigma at baseline			
Yes (Score: 2-6)	52 (62.7)	87 (56.5)	0.359
No (Score: 0-1)	31 (37.3)	67 (43.5)	
Depressive symptoms at baseline			
Yes (Score: 13-33)	50 (62.5)	92 (58.6)	0.562
No (Score: 0-12)	30 (37.5)	65 (41.4)	

Pearson’s chi-square test.

### Demographic characteristics of patients on ART

Seventy-four (47.1%) of patients visited the district hospital for ART services (Table 2). The median age was 35 years old (range: 18–68), 94 (59.9%) were female, and 105 (66.9%) were married. Around half of patients (n = 77, 49.4%) had not completed the standard primary education of seven years and 107 (68.2%) were farmers. Sixty-one (38.9%) reported experiences of food insufficiency in the previous 30 days.

**Table 2:** Sociodemographic, physical mental health characteristics of patients who were adherent and non-adherent to ART

	**Total**	**Not full adherence**	**Full adherence**	
	**(n=157)**	**(n=63)**	**(n=94)**	**p-value**
	**n(%)**	**n(%)**	**n(%)**	
Site				
Mumbwa District Hospital	74 (47.1)	32 (50.8)	42 (44.7)	0.452
Rural Health Centers	83 (52.9)	31 (49.2)	52 (55.3)	
Age				
<35 years old	70 (46.1)	25 (41.7)	45 (48.9)	0.381
≥35 years old	82 (53.9)	35 (58.3)	47 (51.1)	
Sex				
Female	94 (59.9)	30 (47.6)	64 (68.1)	0.010*
Male	63 (40.1)	33 (52.4)	30 (31.9)	
Education				
No or primary incomplete	77 (49.4)	30 (48.4)	47 (50.0)	0.844
Primary complete or more	79 (50.6)	32 (51.6)	47 (50.0)	
Marital status				
Married	105 (66.9)	45 (71.4)	60 (63.8)	0.321
Not married	52 (3.1)	18 (28.6)	34 (36.2)	
Occupation				
Agriculture	107 (68.2)	26 (41.3)	24 (25.5)	0.038*
Others	50 (31.8)	37 (58.7)	70 (74.5)	
Transportation				
On foot	80 (51.0)	36 (57.1)	44 (48.8)	0.204
Others	77 (49.0)	27 (42.9)	50 (53.2)	
Time required for transportation to health facilities by above method				
Within one hour	70 (45.2)	29 (46.8)	41 (44.1)	0.742
More than one hour	85 (54.8)	33 (53.2)	52 (55.9)	
Transportation cost				
Zero	127 (80.9)	50 (79.4)	77 (81.9)	0.690
More than zero	30 (19.1)	13 (20.6)	17 (18.1)	
Lack of food during the past 30 days				
Yes	61 (38.9)	14 (22.2)	47 (50.0)	<0.001***
No	96 (61.1)	49 (77.8)	47 (50.0)	
Knowing HIV status				
Within 30 days	65 (41.7)	37 (39.8)	28 (44.4)	0.562
More than 30 days	91 (58.3)	56 (60.2)	35 (55.6)	
Functional status				
Working	138 (87.9)	58 (92.1)	80 (85.1)	0.190
Ambulant or bed lid	19 (12.1)	5 (7.9)	14 (14.9)	
Tuberculosis				
Positive	11 (7.1)	5 (7.9)	6 (6.5)	0.722
Negative or not sure	145 (92.9)	58 (92.1)	87 (93.5)	

Pearson’s chi-square test; *p<0.05;***p<0.001.

With regard to the transportation, 127 (80.9%) did not need to pay the transportation fee to access the district hospital or rural health centers. Over half of patients (n = 80, 51%) travelled to the health facility on foot and 85 (54.8%) required more than one hour for this journey. The general physical and mental health of patients was also surveyed. One hundred thirty-eight (87.9%) had a functional status of being able to work, and 11 (7.1%) were smear**-**positive for TB. Over half of patients had self-stigma (n = 87, 56.5%) and were depressed (n = 92, 58.6%). In addition, 89 (85.6%) disclosed their HIV status to their spouse, and 36 (29.3%) spouses were also receiving ART (Table 3).

**Table 3:** Interpersonal characteristics of patients who were adherent and non-adherent to ART

	**Total**	**Not full adherence**	**Full adherence**	
	**(n=157)**	**(n=63)**	**(n=94)**	**p-value**
	**n(%)**	**n(%)**	**n(%)**	
Disclose to First wife/husband if married				
Yes	89 (85.6)	35 (77.8)	54 (91.5)	0.048 ^a*^
No	15 (14.4)	10 (22.2)	5 (8.5)	
First additional sexual partner				
Yes	13 (59.1)	7 (63.6)	6 (54.5)	1.000 ^b^
No	9 (40.9)	4 (36.4)	5 (45.5)	
People in community know my HIV status even though I did not tell them				
Yes	48 (38.6)	21 (37.5)	27 (31.0)	0.424 ^a^
No or unsure	95 (66.4)	35 (62.5)	60 (69.0)	
First wife/husband’s HIV status if married (including divorced, separated, widow/widower)				
Positive	58 (44.6)	20 (37.7)	38 (49.4)	0.190 ^a^
Negative or unsure	72 (55.4)	33 (62.3)	39 (50.6)	
First wife/husband is (was) on ART (including divorced, separated, widow/widower)				
Yes	36 (29.3)	10 (20.0)	26 (35.6)	0.062 ^a^
No or unsure	87 (70.7)	40 (80.0)	47 (64.4)	
First additional sexual partner’s HIV status				
Positive	0 0.0	0 0.0	0 0.0	
Negative or unsure	21 (100.0)	10 (100.0)	11 (100.0)	
First additional sexual partner is on ART				
Yes	0 0.0	0 0.0	0 0.0	
No or unsure	18 (100.0)	9 (100.0)	9 (100.0)	

a: Pearson’s chi-square test; b: Fisher’s exact test; *p<0.05.

### Status of ART adherence

Among 157 patients on ART who were included in this study, 94 (59.9%) were fully adherent and 63 (40.1%) were non-adherent to their treatment six weeks after starting ART.

### Factors associated with full ART adherence

Bivariate analysis indicated that being female (*p* = 0.010), disclosure to spouses (*p* = 0.048), and experiences of food insufficiency in the previous 30 days (*p* < 0.001) were positively associated with adherence to ART, but being farmers (*p* = 0.038) were negatively associated (Tables 2 &3). Additionally, the proportion of patients who were adherent to ART was likely to be higher among patients whose spouse was also receiving ART than patients whose spouse was not, although this was not statistically significant (*p* = 0.062) (Table 3).

To identify the factors associated with full ART adherence, multiple logistic regression analysis was performed. Full adherence was associated with being female [AOR, 3.3; 95% Confidence interval (CI), 1.2 - 8.9], having a spouse who were also receiving ART (AOR, 4.4; 95% CI, 1.5 - 13.1) and experience of food insufficiency in the previous 30 days (AOR, 5.0; 95% CI, 1.8 - 13.8) (Table 4).

**Table 4:** Multiple logistic regression analysis of factors affecting adherence to ART (n=96)

**Variable**	**B**	**SE**	***β***	**p**	**AOR**	**95% CI**
Gender (Females)	0.21	0.09	0.21	0.021*	3.26	1.20-8.90
Experience of food insufficiency in the previous 30 days	0.30	0.09	0.31	0.002**	5.00	1.81-13.76
Disclose HIV status to spouse	0.20	0.12	0.15	0.130	2.85	0.73-11.06
Spouse on ART	0.26	0.09	0.25	0.007**	4.44	1.50-13.12
R^2^	0.28					

B: Unstandardized coefficient; SE: Standard error; *β*: Standardized coefficient; p value at <0.05* & <0.01**; A0R: Adjusted odds ratio; CI: Confidence interval; R^2^: Coefficient of determination. The variables of which the associated p value level was less than 0.1 by an univariate analysis were entered into a multiple logistic regression model.

### Reasons for missed doses

Among patients who were not adherent to ART (n=63), 39 gave reasons for missed doses. Some of the most common reasons were long distance to health facilities (n = 21, 53.8%), food insufficiency (n = 20, 51.3%), being busy with other activities such as work (n = 15, 38.5%), being depressed (n = 5, 12.8%), and forgetfulness (n = 4, 10.3%) (Table 5).

**Table 5:** Reasons for missed doses (multiple answers) (n=39)

	**n(%)**
Long distance to health facilities	21 (53.8)
Not enough food	20 (51.3)
Were busy with other things like work etc.	15 (38.5)
Felt depressed, overwhelmed, hopelessness	5 (12.8)
Forgot	4 (10.3)
Felt sick or ill	3 (7.7)
Ran out of pills	2 (5.1)
Traditional prohibition	2 (5.1)
Felt asleep or slept through dose tine	1 (2.6)
Had a change in daily routine	1 (2.6)
Had problem taking pills at specific times with meals	1 (2.6)
Sold out the pills	1 (2.6)

## Discussions

### Status of ART adherence in the Mumbwa district

Based on a self-report, 59.9% were considered to be fully adherent to ART six weeks after starting the treatment. The adherence was lower than that found in studies conducted in other countries in Sub-Saharan Africa, which have shown a full medication adherence of 76% [27]. Although a simple comparison is not accurate because of large variation between the surveys, the difference could be explained as follows.

First, it could be related to the different approach for measuring ART adherence. As long-term viral suppression requires consistent and high level dose adherence accompanied by optimal inter-dose intervals [28], adherence to dose as well as adherence to schedule was considered in this study. Those who never skipped prescribed drugs and followed time restrictions during the previous four days were considered to be fully adherent. In contrast, most other studies that have measured ART adherence using patient self-reporting have only looked at adherence to dose instructions [27].

Second, possible difference in access to treatment among patients who were in need for ART in Zambia and other countries in Sub-Saharan Africa could also have influenced the result. Nearly 70% of those in need for treatment have accessed it in Zambia in both urban and rural areas. On the other hand, studies in other African countries were conducted mostly in urban areas [27] where patients have relatively easy access to health facilities. It could be expected that relatively high levels of adherence in other studies may decline as treatment access expands to rural area where people have poor access to the health facility.

### Factors related to full adherence to ART

In a multivariate analysis, gender remained a significant factor after adjusting for potential confounding variables. In a study in rural Uganda, female patients had a significantly higher CD4 cell count at the initiation of ART and lower mortality six months later than male patients, as female patients had more opportunities to access care and start treatment at less advanced stages of HIV, potentially through their participation in prevention of mother-to-child transmission (PMTCT) programs [29]. In Zambia, PMTCT initiative was launched in 1999 and expanded such that an estimated 69% of pregnant women living with HIV had received antiretroviral (ARV) drugs for PMTCT by the end of 2009 [30]. As the case of Uganda, national PMTCT services in Zambia may have contributed to earlier access to ART and supported better ART adherence for a larger group of HIV-positive female, since early ART results in less AIDS progression and death with no increase in adverse events or loss of virologic response compared to deferred ART [31].

Although not determined in this study, sex differences in treatment response and side effects could also have contributed to the outcomes observed in this study. Another study showed that female patients had better responses to treatment compared with that of male patients, and side effects related to ARV drugs were more frequently observed in male patients than in female patients [32].

Additionally, female patients may have had greater motivation to adhere to ART as suggested by other study that female patients caring for children emphasize their role as primary care to children and their children are known to be a facilitating factor in adherence among them [33].

However, other studies have also shown that HIV-positive females often experience gender-related barriers to accessing health services, thus affecting ART adherence [34,35]. For example, many females have to obtain permission from a male spouse or a relative to seek HIV care, which is difficult when females have to ask for money and take time away from household chores. In addition, where costs for treatment are involved, families may prioritize paying for male’s treatment [34]. Gender-based violence has also affected female’s access and ART adherence [36]. Although these barriers were not found in this study, the issue demands further exploration, particularly given the different social influences on male and female [37].

Regarding HIV disclosures, over 80% disclosed their status to their spouse. This was positively associated with treatment adherence in bivariate analysis, although it was not seen in the final analysis. The spousal disclosure rate was relatively higher in this study than in a study conducted in another part of rural Zambia in 2005–2006 [12]. The higher rate of disclosure in this study may be attributed to the establishment of peer counselors or treatment supporters during the past few years in Zambia, which encourages disclosure and treatment adherence in the district hospital and rural health centers. However, disclosure could still have both positive and negative consequences. Disclosure has the potential to yield much-needed social support. Alternatively, it may also lead to stigmatization, discrimination, abandonment or gender-based violence mentioned above after disclosing their HIV status to their spouse or partners. Strategies are needed to take account of HIV positive patients who want to disclose HIV status safely to their surroundings.

Having a treatment partner such as a spouse, family member, friends or peer counselor is known to be positively associated with ART adherence [38]. In this study, having a spouse who is also on ART was found to be positively associated with ART adherence. Spouses on ART might play a role as a treatment partner more readily than spouses not on ART because they have a better understanding of treatment adherence for their partners and themselves. While it could not be determined from the results whether spouses were supportive, ART programs should consider the potential benefit of treatment support provided by persons close to patients, especially from those on ART.

A number of qualitative studies have reported that food insufficiency is an important barrier to ART adherence [15,16,39,40]. In urban Peru, Franke et al. [26] found that individuals who reported food insufficiency in the month prior to interview were more likely to experience suboptimal adherence than those who did not. In three rural ART clinics located in another part of rural Zambia, patients who had skipped a meal because of a lack of food in the past week were more likely to have poor adherence [41].

However, on the contrary, this study found that the experience of food insufficiency in the previous 30 days from the baseline interview date was positively associated with treatment adherence. This may be explained by enhanced social support targeting people living in such extreme poverty that they cannot afford to buy food described as below. A pilot study in Zambia found that individuals with food insufficiency who received nutritional support demonstrated significantly better ART adherence compared with a group who did not receive this support [13]. The World Food Program (WFP) has been implementing a program in Zambia since 1967 and is committed to providing food assistance to approximately 2.3 million people in Zambia in 2011 [42]. Patients who were extremely poor and suffered from insufficient food at the initiation of ART might more easily receive such assistance compared with patients who were not so poor that they ever experienced food insufficiency at the time, although it is difficult to rely only on such explanation to account for everything since some donors have suspended their assistance in Mumbwa.

On the other hand, one of the most cited reasons for missing doses by this study population was also ‘food insufficiency’, and the experience of food insufficiency in the previous 30 days from the follow-up interview date was also associated with poor treatment adherence by bivariate analysis (data not shown). This paradoxical finding could be understood by considering the timing of the interviews. The question about reasons for missing doses was asked six weeks after starting ART, while the positive association was found between treatment adherence and the experience of food insufficiency in the previous 30 days from the baseline interview date. Therefore, patients who experienced food insufficiency in the 30 days previous to ART initiation might have received food supplementation or counseling afterwards, which might have supported their adherence positively as mentioned above. However, patients who were not so poor thus they could not receive food assistance at the initiation of ART might have needed financial assistance after starting the treatment because of transportation fee and loosing wages due to long waiting times for a clinic visit. They might eventually have fallen into poverty and food insecurity after starting ART, and could not adhere to treatment, even if the ART services are provided free of charge in Zambia.

Moreover, some patients may have been taught and believed that ARV drugs always need to be taken with food and some might have missed the medication as they missed their meal due to the food insufficiency. Thus, individuals who missed ARV drugs might consider food insufficiency the reason. However, more studies are needed to better understand the association between ART adherence and food issues, because a single question was used to assess food insufficiency in this study, which is only one aspect of the issues.

In addition to ‘food insufficiency’, the frequently cited reasons for missing doses in this study were ‘long distance to health facilities’ and ‘being busy with other things like work’. This observation is consistent with findings in other studies [16,18,27,39,43-54].

Although time required for transportation to the health facilities and transportation fees were not significantly associated with treatment adherence, accessing to treatment facilities can be a problem for many patients living in Mumbwa. This is supported by the result that it took over one hour to reach health facilities in half of patients on ART in this study. Because of this long distance to access to health facilities in Mumbwa, patients who missed doses might report that the distance from home to the district hospital or rural health centers caused the disruption in ART adherence.

In addition, patients whose occupation was agriculture were more likely to have poor adherence. It is possible that patients who worked in agriculture had difficulty coming for health facilities because of their work’s seasonal nature. This is probably supported by our finding that ‘being busy with other things like work’ was the major reason for missing doses.

To enhance understanding of self-stigma and depressive symptoms among patients on ART in Zambia, the associations between these psychological factors and treatment adherence were assessed. Although these factors have been identified as factors associated with poor adherence in multiple studies, no associations were found in this study. The limited numbers of subjects with poor adherence may have prevented the identification of potential associations. More work is needed to investigate patients’ self-stigma and depressive symptoms with having to adhere to lifelong regimens.

### Limitations

This study has several limitations. First, assessment of treatment adherence based on a self-report may be subject to recall and social desirability bias that may result in under-reporting of missed pill intakes. Thus, an over-estimation of adherence is possible. However, there is evidence that a simple self-report adherence questionnaire provides a sensitive measure of non-adherence that predicts viral rebound and is almost always reliable [55-57]. It is also an inexpensive and quick method to use in a field research and resource poor setting.

Second, we could only include those individuals who initiated ART at the target health facilities, and returned six weeks later. Hence, we may also have slightly over-estimated the actual adherence levels of this population, and our sample may not be enough to detect significant associations between patients who were adherent to ART six weeks after starting the treatment and who were not. However, this influence is likely to be limited because there were no significant differences in basic sociodemographic or health characteristics between patients who were included or excluded in this study except for the required time to access health facilities (Table 1).

Third, in relation to recent changes in Zambia, this study was conducted in a rural area where ART services have been initiated. Therefore, it is difficult to generalize the study findings to the population in areas where ART services are not yet available, although there were no differences in patients’ characteristics between those who visited the district hospital and rural health centers.

### Future challenges

This study investigated patients’ adherence to ART over a short period of time, because the initial response to ART has long-term prognostic significance, and optimizing adherence in the early months is important for ensuring long-term immunological and virological success [19,20]. However, long-term analyses are clearly needed to fully assess factors related to treatment adherence and to allow some generalizability of the results.

In addition, one of the most cited reasons for missing doses was long distance to health facilities, and only difference in basic sociodemographic or health characteristics between patients who were included and excluded in this study was required time to access health facilities. It is therefore necessary to examine the association between travel-related variables and adherence to ART in detail in future research, although they did not predict the adherence in another study in rural Zambia [14].

Finally, recent changes such as the adoption of free access to ART in Zambia may have some implications for the study results. While it is expected that this policy will reduce financial constraints, the high level of other health expenditures still experienced by patients suggests that the detrimental influence of out-of-pocket payments will certainly not be fully eliminated. Further studies are needed to assess and examine this policy for treatment adherence and its interruptions over longer time scales.

## Conclusions and recommendations

Social supports from spouses and people on ART could facilitate their adherence to ART. This is likely to require attention by ART services in the future, focusing on different social influences on male and female in rural Zambia. In addition, poverty reduction strategies could help to reinforce adherence to ART and mitigate the influence of HIV infection for poor patients and those who fall into poverty after starting ART.

## Competing interests

The authors declare that they have no competing interests.

## Authors’ contributions

YS, KKa, SM, NI, KKi and IK carried out data analysis and drafted this manuscript. YS, CD, IS, CM, GS, KKo, SM and NI helped to collect data and participated in coordinating the study design to involve trained interviewers. KKa, KKi and IK helped with the design of this study. All authors read and approved the final manuscript.
